# Morphology Control of Zr-Based Luminescent Metal-Organic Frameworks for Aflatoxin B1 Detection

**DOI:** 10.3390/bios14060273

**Published:** 2024-05-27

**Authors:** Fang Zhu, Qiuxue Chai, Dinghui Xiong, Nuanfei Zhu, Jialong Zhou, Ruoxi Wu, Zhen Zhang

**Affiliations:** School of the Environment and Safety Engineering, School of Emergency Management, Jiangsu University, Zhenjiang 212013, China; zhufang@ujs.edu.cn (F.Z.); chaiqiuxue@163.com (Q.C.); xdh602422@163.com (D.X.); zhunuanfei@163.com (N.Z.); zhoujialong2000@163.com (J.Z.); 3210904003@stmail.ujs.edu.cn (R.W.)

**Keywords:** sensors, metal–organic frameworks, rapid detection, aflatoxin B1, fluorescence

## Abstract

Metal–organic frameworks (MOFs) have gained significant prominence as sensing materials owing to their unique properties. However, understanding the correlation between the morphology, properties, and sensing performance in these MOF-based sensors remains a challenge, limiting their applications and potential for improvement. In this study, Zr-MOF was chosen as an ideal model to explore the impact of the MOF morphology on the sensing performance, given its remarkable stability and structural variability. Three luminescent MOFs (namely rod-like Zr-LMOF, prismoid-like Zr-LMOF, and ellipsoid-like Zr-LMOF) were synthesized by adjusting the quantities of the benzoic acid and the reaction time. More importantly, the sensing performance of these Zr-LMOFs in response to aflatoxin B1 (AFB1) was thoroughly examined. Notably, the ellipsoid-like Zr-LMOF exhibited significantly higher sensitivity compared to other Zr-LMOFs, attributed to its large specific surface area and pore volume. Additionally, an in-depth investigation into the detection mechanism of AFB1 by Zr-LMOFs was conducted. Building upon these insights, a ratiometric fluorescence sensor was developed by coordinating Eu^3+^ with ellipsoid-like Zr-LMOF, achieving a remarkably lower detection limit of 2.82 nM for AFB1. This study contributes to an improved comprehension of the relationship between the MOF morphology and the sensing characteristics while presenting an effective approach for AFB1 detection.

## 1. Introduction

Metal–organic frameworks (MOFs) consisting of metal ions/clusters and organic linkers have been extensively studied in the fields of gas storage, catalysis, drug delivery, and energy storage [1,2,3,4,5,6]. Recently, the utilization of MOFs as sensor materials has attracted significant attention due to their modular synthesis, advanced functional properties, and improved sensing efficiency in detecting analytes [7,8,9,10]. For instance, Zeng et al. synthesized mixed lanthanide metal–organic frameworks that enable the rapid and highly sensitive optical detection of fluoride ions [11]. Additionally, Che et al. fabricated the fluorescent film based on Eu-based MOF for the swift detection of formaldehyde [12].

Due to their definite compositions and periodic backbones, MOFs exhibit structure-dependent properties that have been detected and applied in various fields. Most representatively, three zinc-porphyrin MOFs showed distinct selective adsorption capacities for various organic dyes by introducing versatile auxiliary ligands [13]. Zhou et al. confirmed topology-dependent chemical stability in PCN-series MOFs under acidic/basic treatments [14]. Similarly, the detection mechanism and sensing performances of MOF-based sensors typically rely on the morphology-dependent properties of MOFs [15]. However, the potential correlation between the morphology, properties, and sensing performance remains poorly understood for these MOF-based sensors, hindering their application in detection and performance improvement [16,17,18]. Consequently, systematic investigations are imperative to establish the link between the MOF morphology and its corresponding detection capabilities.

Zr-MOFs have been recognized as ideal models for investigating the relationship between the MOF morphology and sensing performance, owing to their extremely high thermal, mechanical, and chemical stability [19,20,21,22,23,24]. Additionally, one of the most attractive features of Zr-MOFs is their morphological diversity, allowing the construction of diverse crystal morphology using the same building blocks [25,26,27,28]. It was demonstrated the adjustments in pH, temperature, reaction time, and the functionalization of organic linkers influence the synthesis of Zr-MOFs. In addition, the synthesis can be modulated by certain modulators, such as acetic and benzoic acid [29]. These strategies enable the synthesis of Zr-MOFs with variable topologies, compositions, and morphologies, providing opportunities for the rational regulation of MOF properties to achieve enhanced sensing performance.

In this study, the Zr^4+^ clusters and TCPB^4−^ were utilized as the building blocks and organic linkers to synthesize the luminescent MOFs. By adjusting the quantity of benzoic acid and the hydrothermal reaction time, three MOFs with distinct morphologies were obtained, being designated as prismoid-like Zr-LMOF, rod-like Zr-LMOF, and ellipsoid-like Zr-LMOF, respectively. The impact of these varied MOF morphologies on the detection of AFB1 was systematically investigated. On this basis, a straightforward one-pot synthesis method was employed to functionalize Zr-LMOF with Eu^3+^, enabling the generation of a ratiometric fluorescence signal, which further improved the sensing performance for AFB1. The study not only developed a highly sensitive AFB1 sensor but also demonstrated the feasibility of morphological regulation in enhancing the sensing performance.

## 2. Experiments

### 2.1. Synthesis of Zr-LMOFs

Rod-like Zr-LMOF was prepared following established protocols [30]. Specifically, 100 mg of ZrCl_4_, 140 mg of H_4_TCPB, and 5400 mg of benzoic acid were ultrasonically dissolved in 32 mL H_2_O:DMF (1:2) within a 50 mL Teflon vessel. The clear mixture was then placed in the reaction kettle at 120 °C and maintained for 48 h. After cooling to room temperature, the obtained white cloudy liquid was washed once with DMF and twice with methanol before being dried at 60 °C overnight. In the absence of benzoic acid, the amorphous colloid-like polymer was obtained through the same conditions. Conversely, the presence of double benzoic acid resulted in the formation of ellipsoid-like Zr-LMOF. The prismoid-like Zr-LMOF was synthesized under the identical conditions of the rod-like Zr-LMOF, with the reaction time reduced to 6 h.

### 2.2. Synthesis of Zr-LMOF/Eu

The synthesis method for Zr-LMOF/Eu involved a coordination post-synthesis approach. Initially, 0.01 g of Zr-LMOF and 2 mM of Eu(NO_3_)_3_·6H_2_O were dissolved in 50 mL of ethanol and heated at 60 °C for 24 h. After cooling to room temperature, the resulting product was collected and subjected to multiple washes with ethanol. Finally, the white product was dried under vacuum at 60 °C.

### 2.3. Fluorescence Assays

For fluorescence detection, 0.5 mg of Zr-LMOF (Zr-LMOF/Eu) was dispersed in 10 mL of ultrapure water at room temperature. Subsequently, 90 μL of the Zr-LMOF suspension was mixed with 10 μL of the sample solution containing AFB1 in final concentrations of 0.005, 0.0075, 0.01, 0.03, 0.09, 0.27, 0.81, 2.43, 7.29, 10.0, 25.0, and 50.0 μM. After a 10 min incubation period, the fluorescence intensity change at 410 nm was monitored using a microplate reader with an excitation wavelength of 340 nm.

## 3. Results and Discussion

### 3.1. Characterization of Zr-LMOFs

**Figure 1:**
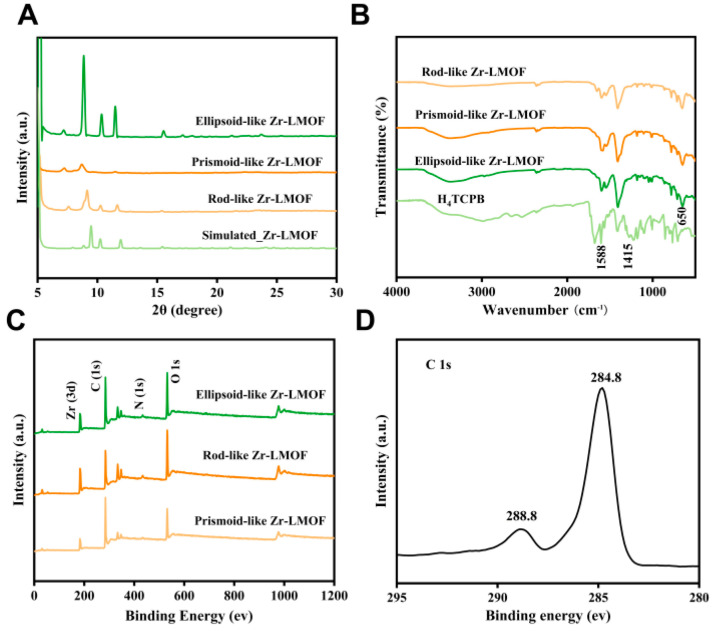
(**A**) Powder XRD pattern of Zr-LMOFs, (**B**) FT-IR spectra of Zr-LMOFs and H_4_TCPB, (**C**) XPS survey spectrum of Zr-LMOFs, and (**D**) XPS spectrum of C 1s regions of ellipsoid-like Zr-LMOF.

Water-stable Zr^4+^ clusters and luminescent TCPB^4−^ serve as second building units and organic linkers, respectively, in the synthesis of Zr-LMOF with benzoic acid acting as the modulator. The resulting Zr-LMOF, as depicted in Figure S1A, exhibits a uniform ellipsoidal shape with sizes ranging from 0.75 to 2.25 μm. Energy dispersive spectroscopy (EDS) elemental mapping reveals the homogeneous distributions of Zr, C, N, and O elements throughout the samples (Figure S1B). In addition, the characteristic diffraction peaks in the X-ray diffraction (XRD) patterns at 10.3° and 11.4° nm align well with prior studies (Figure 1A) [31]. Moreover, the Fourier transform infrared (FT-IR) spectrum was employed to probe the coordination reaction between the H_4_TCPB and Zr-O clusters. It can be seen in Figure 1B that the ellipsoid-like Zr-LMOF spectrum displays a distinctive characteristic peak at 650 cm^−1^ corresponding to the Zr-O cluster. Concurrently, the peak intensity of the carbonyl group decreases at 1588 cm^−1^ and intensifies at 1415 cm^−1^, indicating the coordination interaction between the TCPB^4−^ and Zr-O clusters. To scrutinize the chemical composition and state of elements in the produced Zr-LMOF, an X-ray photoelectron spectroscopy (XPS) analysis was conducted. Figure 1C shows that the Zr-LMOF consisted of four primary elements: Zr, C, N, and O. The C 1s spectrum (Figure 1D) exhibits peaks at 284.8 and 288.8 eV, assigned to C=C and −COOH in TCPB^4−^, respectively. The Zr 3d spectrum (Figure S2) displays distinct peaks for Zr 3d_5/2_ and Zr 3d_3/2_ at 182.6 and 185.0 eV [32,33], respectively, demonstrating the existence of Zr^4+^. All the results affirm the successful synthesis of ellipsoid-like Zr-LMOF.

**Figure 2:**
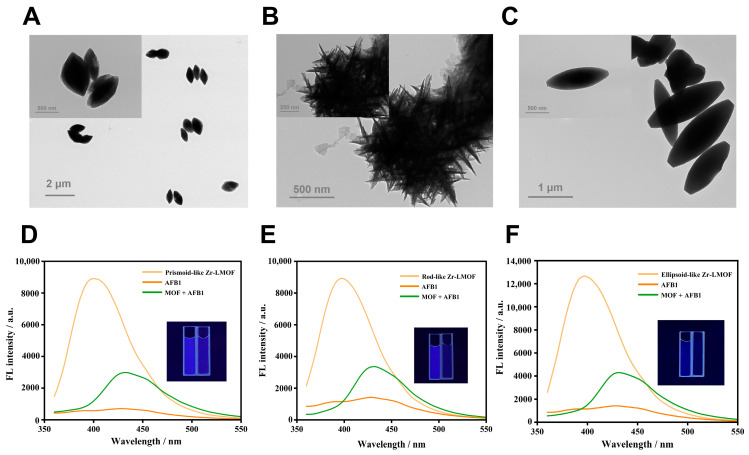
TEM images of (**A**) prismoid-like Zr-LMOF, (**B**) rod-like Zr-LMOF, and (**C**) ellipsoid-like Zr-LMOF. The fluorescence emission spectra of three Zr-LMOFs with 50 μM AFB1 from (**D**–**F**) (the inset shows Zr-LMOF (**left**) with Zr-LMOF + AFB1 (**right**) under 302 nm UV irradiation).

In general, the synthesis conditions intricately govern the shape, size, nucleation, growth rates, and crystallinity of MOFs [34]. Herein, the reaction time and the amount of benzoic acid (as the modulator for controlling the nucleation rate of Zr-LMOFs) were carefully regulated, and distinct samples were prepared. The strong π–π interaction within the framework, as reported by Li et al. [30], induced an absorption peak shift of H_4_TCPB from 280 nm to 316 nm, 395 nm, and 345 nm in the rod-like Zr-LMOF, prismoid-like Zr-LMOF, and ellipsoid-like Zr-LMOF, respectively (Figure S3). For comparison, TEM was used to investigate the surface morphologies of different Zr-LMOFs. It can be seen in Figure 2 that the morphology of the Zr-LMOFs can be modulated via varying the reaction time and the amount of the modulator. Under benzoic acid modulation, a 6-h reaction yielded prismoid-like Zr-LMOF (Figure 2A) with a size of approximately 1 μm. Extending the reaction time to 48 h resulted in rod-like Zr-LMOF crystals, emphasizing the pivotal role of the reaction duration in Zr-LMOFs’ morphology regulation (Figure 2B). On the other hand, doubling the amount of benzoic acid with the same reaction time produced ellipsoid-like Zr-LMOF with lengths ranging from 0.75 to 2.25 μm (Figure 2C). Conversely, in the absence of benzoic acid, an amorphous colloid-like polymer formed, exhibiting limited suspension properties (Figure S4). It suggested the significance of the benzoic acid quantity in controlling the Zr-LMOF synthesis. Additionally, the characteristic diffraction peaks in the XRD of the three Zr-LMOFs matched well with that of the simulated Zr-LMOF (Figure 1A). Importantly, the XRD patterns displayed narrower reflections with increasing amounts of benzoic acid, elucidating the adjustability of the Zr-LMOF grain size through the benzoic acid-to-zirconium chloride ratio [34]. Remarkably, the FT-IR analysis indicated subtle differences in the FT-IR peaks of Zr-LMOFs (Figure 1B), suggesting the minimal impact of the reaction conditions on the formation of the functional groups. Furthermore, the water stability of the three Zr-LMOFs was assessed, revealing excellent stability over 15 days, with ellipsoid-like Zr-LMOF exhibiting the most consistent fluorescence intensity preservation (Figure S5).

### 3.2. Morphology-Dependent Fluorescent Response of Zr-LMOFs toward AFB1

The fluorescent responses toward the AFB1 of different Zr-LMOFs were studied. As the results displayed in Figure 2D–F show, the synthetic materials, except the amorphous colloid-like polymer, show significant fluorescence quenching in the presence of AFB1 (Figure S6). In contrast, the colloid-like polymer shows almost no response to AFB1 (Figure S7). Subsequently, the analytical performances of three kinds of Zr-LMOFs-based sensors were assessed. Since the amount of Zr-LMOFs is essential for the analytical process, it was optimized, and the concentrations with the highest signal-to-noise ratio were used in the subsequent experiments (Figure S8).

**Figure 3:**
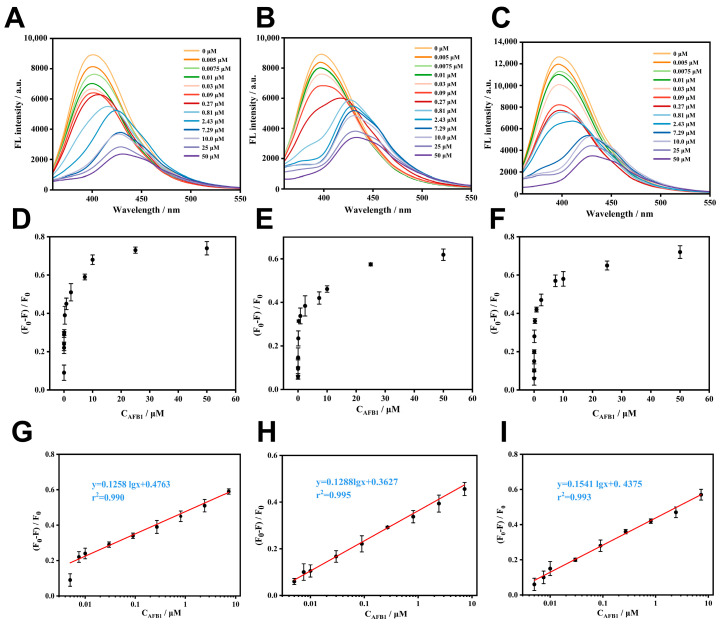
Fluorescence spectra and the corresponding standard curves of three Zr-LMOFs in the presence of different concentrations of AFB1. Fluorescence spectra of (**A**) prismoid-like Zr-LMOF, (**B**) rod-like Zr-LMOF, and (**C**) ellipsoid-like Zr-LMOF and (**G**–**I**) the corresponding calibration curves at low target concentrations from (**D**–**F**), respectively. Fluorescence spectra were recorded with the excitation of 340 nm. Error bars were obtained from three repeats.

Figure 3A–C show the corresponding fluorescence spectra of these sensors at different AFB1 concentrations. For these Zr-LMOFs, a consistent decrease in the fluorescence emission intensity at 410 nm with increasing AFB1 concentrations was observed. In particular, good logarithmic relationships between the AFB1 concentrations and relative fluorescence intensity (F = ∆F/F_0_) were established with the three Zr-LMOFs (Figure 3D–F). The relative fluorescence intensity of the three Zr-LMOFs exhibits a strong linear correlation with the logarithm of AFB1 concentration (Figure 3G–I). According to the developed standard curves, the corresponding detection ranges and detection limits (LODs) were obtained and are summarized in Table S1. The detection limit of the ellipsoid-like Zr-LMOF (3σ/K, where σ is the standard deviation of the blank solution, and K is the slope of the calibration curve) is 5.12 nM, which is lower compared to the rod-like Zr-LMOF (LOD: 12.3 nM) and prismoid-like Zr-LMOF (LOD: 7.39 nM), demonstrating the significant impact of the MOF morphology on the analytical performance. In addition, the AFB1 adsorption experiments were performed on different morphologies of Zr-LMOFs (Table S2). According to the experimental findings, only a small amount of AFB1 was absorbed by the Zr-LMOFs. Furthermore, there was a negligible difference in the adsorption capacity among the three different morphologies of Zr-LMOFs. The selectivity of Zr-LMOF was also explored, as shown in Figure S9, and all the Zr-LMOFs exhibited much weaker responses to the AFB1 analogs. The fluorescence quenching triggered by AFB1 was almost twice as pronounced as that seen with AFB2, AFG1, AFM1, and AFM2. Notably, there were no discernible differences in selectivity observed among these three MOFs.

To investigate the reasons for the difference in the sensitivity among different Zr-LMOFs, Zeta Potential analysis and BET experiments were conducted. As shown in Table S3, the Zeta Potentials of the rod-like Zr-LMOF, prismoid-like Zr-LMOF, and ellipsoid-like Zr-LMOF are −12.5 ± 1.51 mV, −8.24 ± 0.69 mV, and −10.2 ± 0.23 mV, respectively. Since AFB1 carries a negative charge (Table S3), it is reasonable to infer that the electrostatic interactions have no obvious effect on the fluorescence response of different Zr-LMOFs against AFB1. Next, Brunauer–Emmett–Teller (BET) experiments were conducted using N_2_ adsorption/desorption isotherms to study the specific surface areas and pore size distributions of the Zr-LMOFs prepared under different reaction conditions. Rod-like Zr-LMOF and prismoid-like Zr-LMOF showed typical type II isotherms, and ellipsoid-like Zr-LMOF showed typical type I isotherms, indicating the presence of micropores (Figure S10). As shown in Table S4, the Zr-LMOFs exhibited adjustable pore systems with surface areas of 669.44, 992.10, and 1773.74 m^2^/g for the rod-like Zr-LMOF, prismoid-like Zr-LMOF, and ellipsoid-like Zr-LMOF, respectively. The micropores and mesoporous apertures in rod-like Zr-LMOF and prismoid-like Zr-LMOF exhibit slight differences, whereas the pore size of ellipsoid-like Zr-LMOF undergoes noticeable changes, possibly due to surface layer enlargement. It is supposed that the large surface area structures could facilitate the transfer of reactants and products, which can enhance mass transport and thus improve the quenching efficiency. Therefore, the high specific surface areas and pore volume of the synthesized ellipsoid-like Zr-LMOF enhanced its response toward AFB1. 

### 3.3. Mechanism for AFB1 Detection by Zr-LMOF

**Figure 4:**
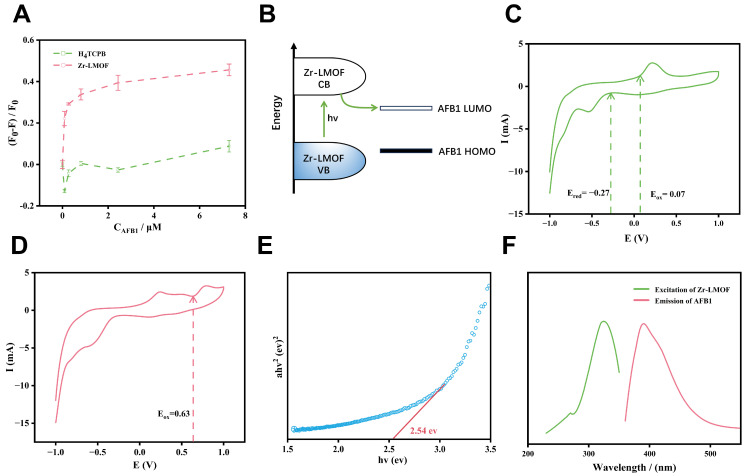
(**A**) FL intensity fading of Zr-LMOF and H_4_TCPB with the existence of AFB1. (**B**) Schematic demonstrating electron transfer from Zr-LMOF to AFB1 LUMO, resulting in quenched emission. (**C**) CV curves of ferrocene tested in 0.1 M tetrabutylammonium hexafluorophosphate solution. (**D**) CV curve of Zr-LMOF. (**E**) Plots of (ahv)^2^ versus energy (hv) of Zr-LMOF. (**F**) Excitation and emission (λ_ex_ = 340 nm) spectra of Zr-LMOF.

By anchoring the TCPB^4−^ with no obvious response to AFB1 into the framework, significant fluorescence quenching appears in the presence of AFB1 (Figure 4A). Therefore, the inclusion of TCPB^4−^ into the long-range ordered structure of the MOF is critical for the sensitive and selective sensing of AFB1. Furthermore, Zr-LMOF exhibits a higher fluorescence intensity than TCPB^4−^, suggesting a higher energy transfer efficiency within the Zr-LMOF (Figure S11).

The quenching of the Zr-LMOF emission by AFB1 is likely due to an electron transfer mechanism that Pramanik et al. previously discussed for LMOF-based sensors [35]. If the lowest unoccupied molecular orbital energy level (LUMO) of electron-rich Zr-LMOF is higher than that of the analyte, the excited electrons of Zr-LMOF can move to the analyte’s LUMO through electron transfer, leading to quenching (Figure 4B). To validate it, electrochemical cyclic voltammetry was performed to assess the energy levels of Zr-LMOF and AFB1 (Figure 4C–E). The highest occupied molecular orbital (HOMO) and LUMO of ellipsoid-like Zr-LMOF were −4.70 eV and −2.12 eV, while those of AFB1 were −11.83 eV and −9.68 eV [36] (Table S5). Therefore, electrons from Zr-LMOF in an excited state can transfer to the LUMO of AFB1, resulting in fluorescence quenching. Energy transfer often contributes significantly to fluorescence quenching and should also be considered. As can be seen in Figure 4F, the spectral overlap between the AFB1 absorption and Zr-LMOF emission is very limited, which hinders the energy transfer from Zr-LMOF to AFB1, indicating that it does not likely play a role in mycotoxin detection.

### 3.4. Radiometric Fluorescence Detection of AFB1 by Zr-LMOF/Eu

To enhance the sensing performance, utilizing ratiometric sensors based on MOFs with multiple emission centers has proven to be a potent strategy [37,38,39]. This approach capitalizes on the self-calibrating mechanism derived from distinct yet interconnected emission centers, effectively minimizing external influences [40,41,42]. Consequently, a ratiometric fluorescent sensor was constructed by incorporating Eu^3+^ into the ellipsoid-like Zr-LMOF, enhancing both its selectivity and sensitivity.

**Figure 5:**
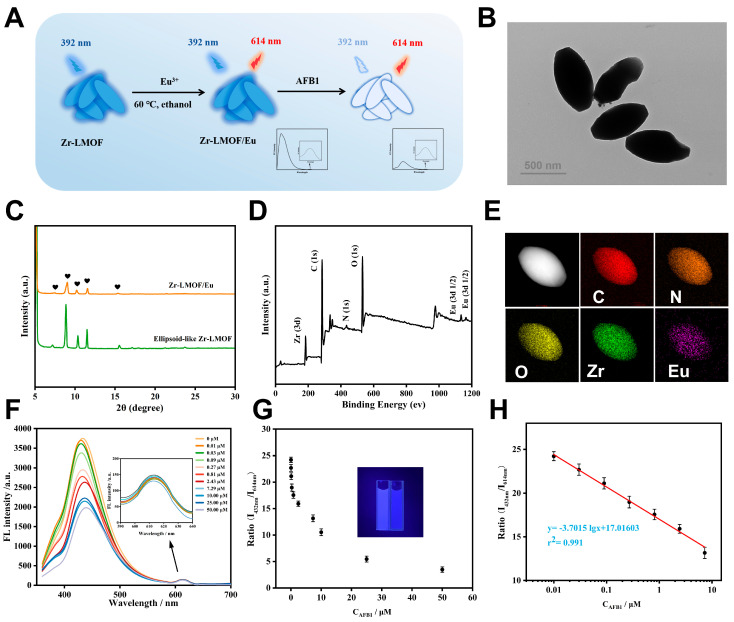
(**A**) Schematic diagram for the application of Zr-LMOF/Eu in AFB1 detection. (**B**) TEM image of Zr-LMOF/Eu. (**C**) Power XRD patterns of Zr-LMOF/Eu. (**D**) XPS survey spectrum of Zr-LMOF/Eu. (**E**) EDS elemental mapping images of Zr-LMOF/Eu. (**F**) Fluorescence emission spectra of Zr-LMOF/Eu with different concentrations of AFB1 (0–50 μM). (**G**) Corresponding relationship and (**H**) standard curve between the ratio of I_432 nm_/I_614 nm_ and the AFB1 concentration.

As shown in Figure 5A, Eu^3+^ was attached to the surface of Zr-LMOF through carboxyl group coordination. Both the TEM images and PXRD spectrum indicate that no phase transition or framework collapse occurred after the modification with Eu^3+^ (Figure 5B,C). Meanwhile, the results obtained from the XPS and EDS elemental mapping analysis confirm the existence and uniform distribution of C, N, O, Zr, and Eu, indicating the successful preparation of Zr-LMOF/Eu (Figure 5D,E). More importantly, the fluorescence intensity at 432 nm varies with the concentration of AFB1, while the fluorescent emission at 614 nm, originating from Eu^3+^, remains constant (Figure 5F). The ratio of I_392 nm_/I_614 nm_ was used as the fluorescence signal for constructing the ratiometric sensor. Figure 5G shows the corresponding relationship between the ratio of I_432 nm_/I_614 nm_ and the logarithm of the AFB1 concentration. The developed ratiometric fluorescent sensor demonstrates a linear detection range spanning from 0.01 μM to 7.29 μM (Figure 5H) and achieves a LOD of 2.82 nM. Remarkably, this LOD is 1.8 times lower than that of the ellipsoid-like Zr-LMOF and either superior or comparable to the majority of previously reported fluorescence sensors designed for detecting AFB1 (Table S1). The selectivity of Zr-LMOF/Eu was also assessed, as depicted in Figure S12, demonstrating that Zr-LMOF/Eu maintains good selectivity for AFB1. Moreover, the method was successfully applied to analyze actual samples, and its performance was validated using classical ELISA as a reference. The assay results, detailed in Table S6, demonstrate outstanding reproducibility (RSD ranging from 1.3% to 4.2%) and accuracy (recoveries ranging from 91.0% to 117.0%). These results align remarkably well with those obtained through ELISA, confirming the accuracy and reliability of the established approach for AFB1 determination.

## 4. Conclusions

In summary, three different morphologies of luminescent metal–organic frameworks with excellent fluorescent properties were synthesized by adjusting the amount of benzoic acid and the reaction time. The impact of these Zr-LMOFs with different morphologies on the sensing performance against AFB1 was thoroughly examined. Notably, the ellipsoid-like Zr-LMOF characterized by its high specific surface areas and pore volume, exhibited an enhanced response to AFB1 in comparison to other morphologies. In addition, the detection mechanism of Zr-LMOF for AFB1 was investigated. It was demonstrated that Zr-LMOF was quenched by AFB1 through facilitating electron transfer due to its higher LUMO energy state. Furthermore, a ratiometric fluorescent sensor for AFB1 was constructed to minimize external influences and achieve greater analytic performance by coordinating Eu^3+^ with ellipsoid-like Zr-LMOF. Our research not only advances our understanding of the relationship between MOF morphology and sensing capabilities but also presents a valuable strategy for AFB1 detection. 

## Data Availability

Data will be made available on request.

## Supplementary Materials

The following supporting information can be downloaded at: https://www.mdpi.com/article/10.3390/bios14060273/s1, Figure S1. (A) TEM image and (B) corresponding EDS elemental mapping images of C, N, O, and Zr of Ellipsoid-like Zr-LMOF. Figure S2. XPS spectrum in Zr 3d region of Ellipsoid-like Zr-LMOF. Figure S3. UV absorption spectra of 0.1 mg mL^−1^ (a) Rod-like Zr-LMOF, (b) Prismoid-like Zr-LMOF, (c) Ellipsoid-like Zr-LMOF, and (d) H_4_TCPB using ultrapure water as solvent. UV absorption spectra of 0.1 mg mL^−1^ (a) Rod-like Zr-LMOF, (b) Prismoid-like Zr-LMOF, (c) Ellipsoid-like Zr-LMOF, and (d) H_4_TCPB using ultrapure water as solvent. Figure S4. TEM image of Colloid-like Polymer. Figure S5. Luminescence stability of three Zr-LMOFs suspended in ultrapure water. (A) Prismoid-like Zr-LMOF, (B) Rod-like Zr-LMOF, (C) Ellipsoid-like Zr-LMOF. Figure S6. The fluorescence emission spectrum of Colloid-like Polymer with 50 μM AFB1. The inset shows the corresponding photographs under UV irradiation at 302 nm. Figure S7. FL fading efficiency of three Zr-LMOFs and Colloid-like Polymer with 50 μM AFB1. Figure S8. FL fading efficiency of 50 µM AFB1 with 5, 10, 50, 100, and 150 µg mL^−1^ Zr-LMOFs, respectively. Figure S9. Fluorescence intensity of Zr-LMOFs towards AFB1, AFB2, AFG1, AFM1, and AFM2. (A) Rod-like Zr-LMOF, (B) Prismoid-like Zr-LMOF, (C) Ellipsoid-like Zr-LMOF. Figure S10. Nitrogen adsorption and desorption isotherms measured at 77.3 K. (A) Rod-like Zr-LMOF, (B) Prismoid-like Zr-LMOF, (C) Ellipsoid-like Zr-LMOF. Figure S11. Emission spectra (λ_em_ = 410 nm) of H_4_TCPB and Zr-LMOF. Figure S12. Fluorescence intensity of Zr-LMOF/Eu towards AFB1, AFB2, AFG1, AFM1, and AFM2. Table S1. Comparison of our method with other sensors for AFB1 detection reported in the literatures. Table S2. Liquid adsorption of AFB1 by MOF. Table S3. Zeta potentials of Zr-LMOFs and AFB1. Table S4. BET Surface area calculated by the Multi-Point BET method, Pore Volume and Average Pore Size by BJH adsorption, Most Frequent Pore Diameter by HK/SF method of Rod-like Zr-LMOF, Prismoid-like Zr-LMOF, and Ellipsoid-like Zr-LMOF. Table S5. The calculated HOMO/LUMO energy levels of Zr-LMOF and AFB1. Table S6. Results of our method and ELISA detecting AFB1 in real samples. References [43,44,45,46,47] are cited in the Supplementary Materials.
