# Supplementary material for: Morphology Control of Zr-Based Luminescent Metal-Organic Frameworks for Aflatoxin B1 Detection
Source: Biosensors (Basel). 2024 May 27;14(6):273. doi: 10.3390/bios14060273 (PMC11201970; doi:10.3390/bios14060273)
Supplement: Supplementary file 1 [file biosensors-14-00273-s001.zip › biosensors-2988875-supplementary.pdf]

Supporting Information

Morphology Control of Zr-Based Luminescent Metal-Organic Frameworks for Aflatoxin B1 Detection

Fang Zhu, Qiuxue Chai, Dinghui Xiong, Nuanfei Zhu, Jialong Zhou, Ruoxi Wu and Zhen Zhang *

School of the Environment and Safety Engineering, School of Emergency Management, Jiangsu University, Zhenjiang 212013, China

* Corresponding author:

Email: zhangzhen@ujs.edu.cn

Fax: +86-511-88790955

Table of Contents

1. **Reagents**

   Instruments

2. **Supporting Figures**

   Figure S1. (A) TEM image and (B) corresponding EDS elemental mapping images of C, N, O, and Zr of Ellipsoid-like Zr-LMOF.

   Figure S2. XPS spectrum in Zr 3*d* region of Ellipsoid-like Zr-LMOF.

   Figure S3. UV absorption spectra of 0.1 mg mL^{-1} (a) Rod-like Zr-LMOF, (b) Prismoid-like Zr-LMOF, (c) Ellipsoid-like Zr-LMOF, and (d) H_4TCPB using ultrapure water as solvent. UV absorption spectra of 0.1 mg mL^{-1} (a) Rod-like Zr-LMOF, (b) Prismoid-like Zr-LMOF, (c) Ellipsoid-like Zr-LMOF, and (d) H_4TCPB using ultrapure water as solvent.

   Figure S4. TEM image of Colloid-like Polymer.

   Figure S5. Luminescence stability of three Zr-LMOFs suspended in ultrapure water. (A) Prismoid-like Zr-LMOF, (B) Rod-like Zr-LMOF, (C) Ellipsoid-like Zr-LMOF.

   Figure S6. The fluorescence emission spectrum of Colloid-like Polymer with 50 μM AFB1. The inset shows the corresponding photographs under UV irradiation at 302 nm.

   Figure S7. FL fading efficiency of three Zr-LMOFs and Colloid-like Polymer with 50 μM AFB1.

   Figure S8. FL fading efficiency of 50 μM AFB1 with 5, 10, 50,100, and 150 μg mL^{-1} Zr-LMOFs, respectively.

   Figure S9. Fluorescence intensity of Zr-LMOFs towards AFB1, AFB2, AFG1, AFM1, and AFM2. (A) Rod-like Zr-LMOF, (B) Prismoid-like Zr-LMOF, (C) Ellipsoid-like Zr-LMOF.

   Figure S10. Nitrogen adsorption and desorption isotherms measured at 77.3 K. (A) Rod-like Zr-LMOF, (B) Prismoid-like Zr-LMOF, (C) Ellipsoid-like Zr-LMOF.

   Figure S11. Emission spectra (λ_{em} = 410 nm) of H_4TCPB and Zr-LMOF.

   Figure S12. Fluorescence intensity of Zr-LMOF/Eu towards AFB1, AFB2, AFG1, AFM1, and AFM2.

3. **Supporting Tables**

   Table S1. Comparison of our method with other sensors for AFB1 detection reported in

the literatures.

Table S2. Liquid adsorption of AFB1 by MOF.

Table S3. Zeta potentials of Zr-LMOFs and AFB1.

Table S4. BET Surface area calculated by the Multi-Point BET method, Pore Volume and Average Pore Size by BJH adsorption, Most Frequent Pore Diameter by HK/SF method of Rod-like Zr-LMOF, Prismoid-like Zr-LMOF, and Ellipsoid-like Zr-LMOF.

Table S5. The calculated HOMO/LUMO energy levels of Zr-LMOF and AFB1.

Table S6. Results of our method and ELISA detecting AFB1 in real samples.

4. Supplemental references

Reagents

Zirconium chloride (99.99%), 1,2,4,5-tetrakis(4-carboxyphenyl)benzene (H_4TCPB, 98%), benzoic acid (99.5%), europium nitrate hexahydrate ($Eu(NO_3)_3 \cdot 6H_2O$, 99.99%), and N, N-dimethylformamide (DMF, 99.9%) were procured from Sinopharm Chemical Reagent Co. Ltd. (Shanghai, China). The analytical standards of AFB1, AFB2, AFG1, AFM1, and AFM2 were obtained from Pribolab (Singapore). The Aflatoxin B1 (AFB1) ELISA Kit was procured from Sinopharm Chemical Reagent Co. Ltd. (Shanghai, China). Milli-Q ultrapure water (Millipore, USA; 18 MU cm) was utilized throughout the experiments.

Instruments

The transmission electron microscopy (TEM) analysis was conducted using a JEM-2100 TEM instrument operating at an acceleration voltage of 200 kV. Field emission scanning electron microscopy (FESEM) imaging, color mapping, and energy dispersive spectroscopy (EDS) analyses were performed utilizing the FESEM instrument (JSM-7800F, JEOL, Japan) with a separate EDS detector (JSM-2100F, JEOL, Japan) attached to the instrument. The FESEM instrument offers magnification ranging from 25 X to 1000 KX and operates at an accelerating voltage of 15 kV. Fourier transform infrared (FT-IR) spectra were acquired using a PerkinElmer Spectrum GX spectrometer (PerkinElmer Co., Waltham, MA). X-ray photoelectron spectroscopy (XPS) analysis was carried out employing a Kratos Axis Ultra DLD instrument with monochromatized Al Kα radiation (1486.69 eV) and a concentric hemispherical analyzer working at 15 kV and 10 mA. The pressure within the sample analysis chamber was maintained below 3×10^{-9} Torr. The X-ray beam was directed normally onto the sample, and the detector was positioned at a 45° angle away from the incident direction. X-ray

diffraction (XRD) patterns were obtained using a diffractometer (D8 ADVANCE, Bruker, Germany) with a scan rate of 5° min^{-1} in the 2θ range from 5° to 80°. Automatic specific surface and porosity analyses were conducted using Quantachrome SI (America). Particle size and surface zeta potential were determined using a zeta sizer analyzer (Nano ZS90, Malvern, UK). Fluorescence spectra were recorded with a microplate reader (Infinite M1000 Pro, TECAN, Switzerland), and UV-Vis absorption spectra were obtained using the UV-Vis spectrophotometer (UV-2600, SHIMADZU, Japan) equipped with a 1 cm quartz cuvette holder for liquid samples.

For SEM, EDS, XPS, and XRD analyses, the prepared powder materials were directly utilized. The TEM sample was prepared by depositing the material suspension onto a carbon-coated Cu grid, followed by drying at ambient conditions. For other characterizations, the material suspension was prepared by diluting and sonicating the dried samples before use.

The crystal structure data of the stimulated Zr-LMOF was provided by Lammert et al. [1], which were imported into "Mercury" software to obtain XRD simulated curves.

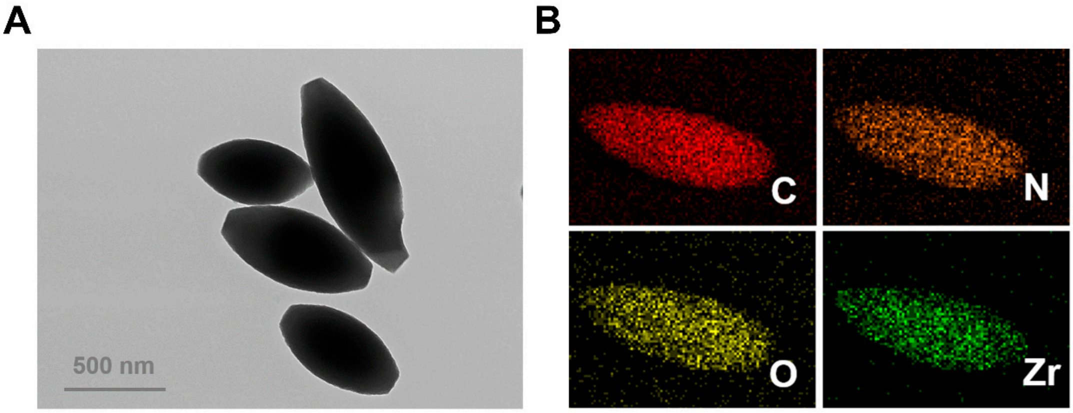

Figure S1. (A) TEM image and (B) corresponding EDS elemental mapping images of C, N, O, and Zr of Ellipsoid-like Zr-LMOF.

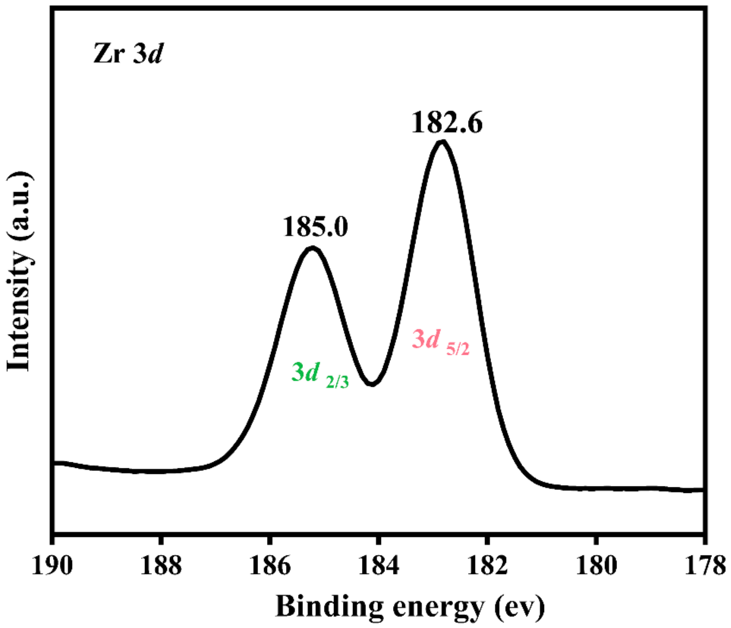

Figure S2. XPS spectrum in Zr 3*d* region of Ellipsoid-like Zr-LMOF.

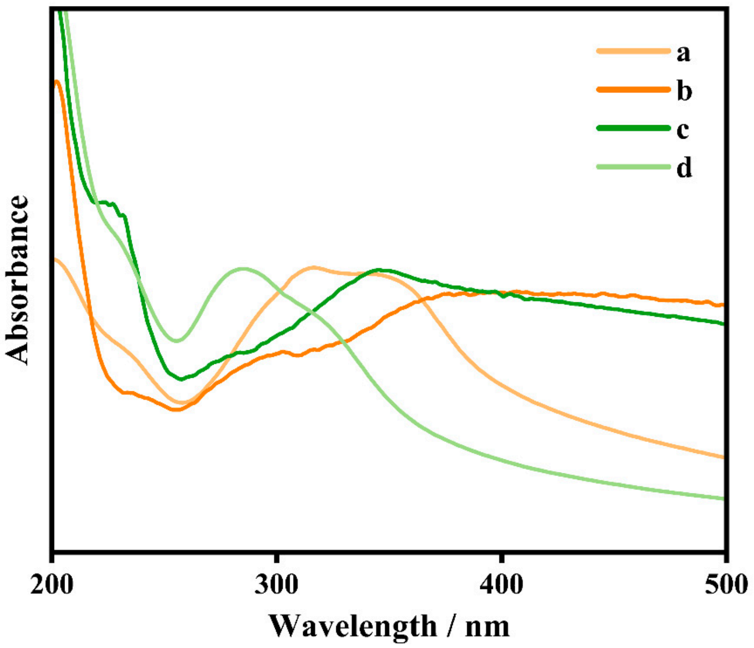

Figure S3. UV absorption spectra of 0.1 mg mL^{-1} (a) Rod-like Zr-LMOF, (b) Prismoid-like Zr-LMOF, (c) Ellipsoid-like Zr-LMOF, and (d) H_4TCPB using ultrapure water as solvent.

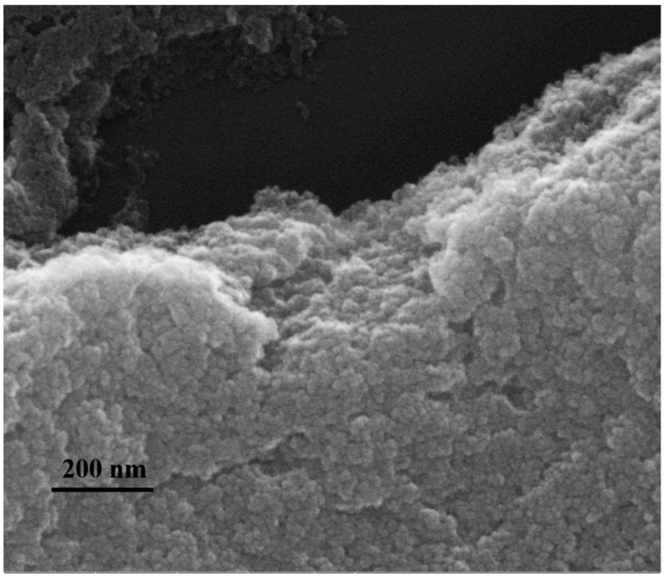

Figure S4. TEM image of Colloid-like Polymer.

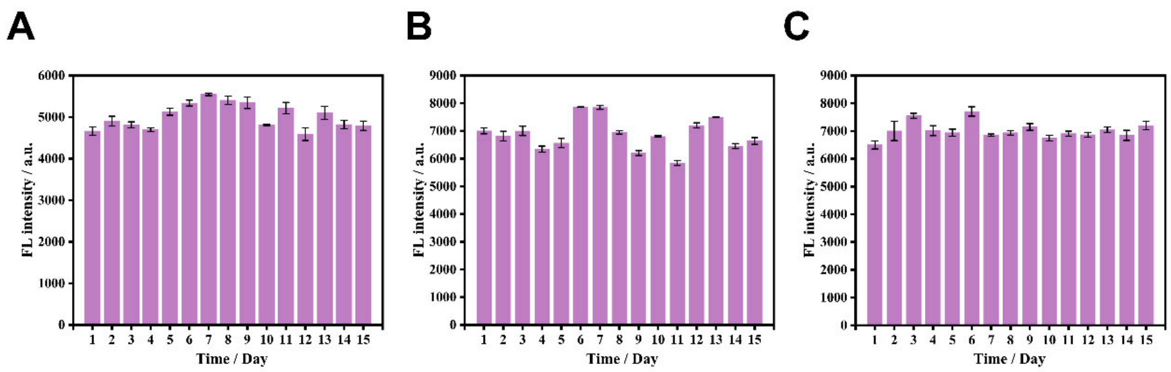

Figure S5. Luminescence stability of three Zr-LMOFs suspended in ultrapure water. (A) Prismoid-like Zr-LMOF, (B) Rod-like Zr-LMOF, (C) Ellipsoid-like Zr-LMOF.

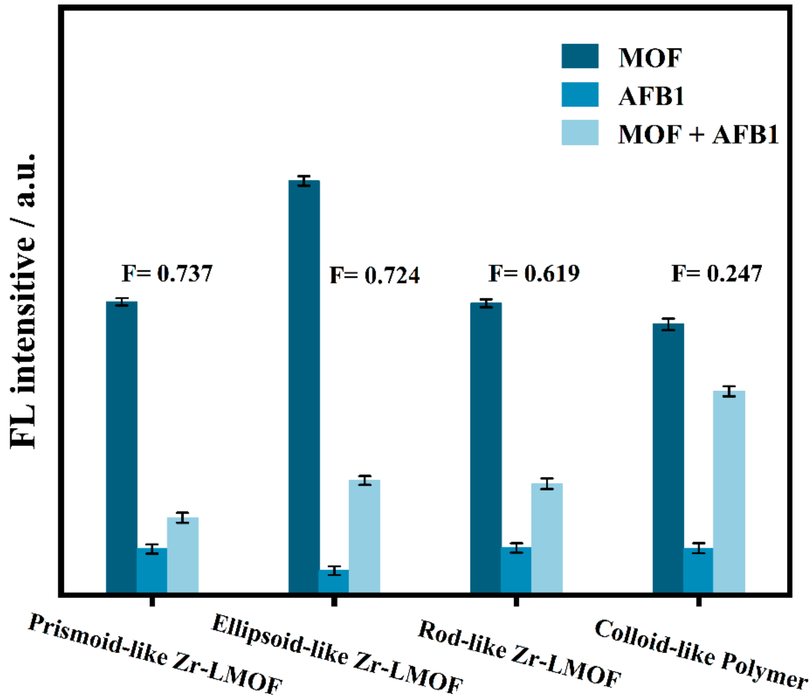

Figure S6. FL fading efficiency of three Zr-LMOFs and Colloid-like Polymer with 50 μM AFB1.

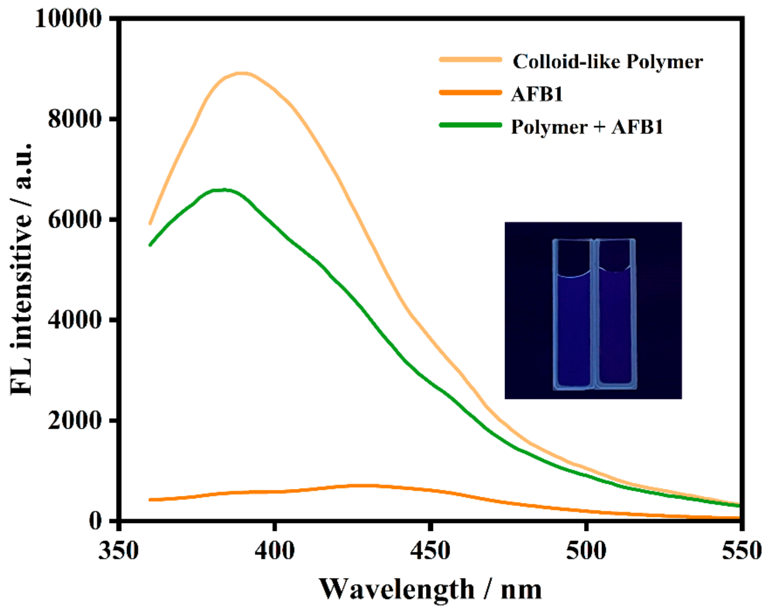

Figure S7. The fluorescence emission spectrum of Colloid-like Polymer with 50 μM AFB1.

The inset shows the corresponding photographs under UV irradiation at 302 nm.

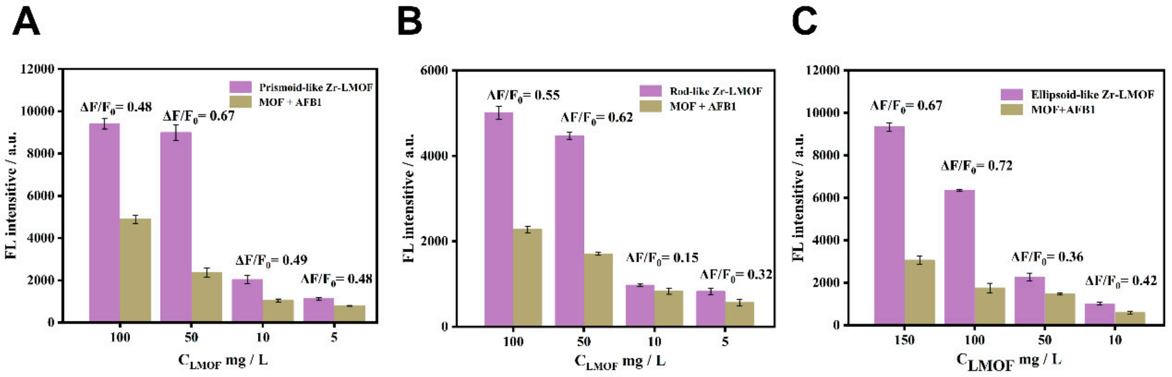

Figure S8. FL fading efficiency of 50 μM AFB1 with 5, 10, 50,100, and 150 μg mL^{-1} Zr-LMOFs, respectively.

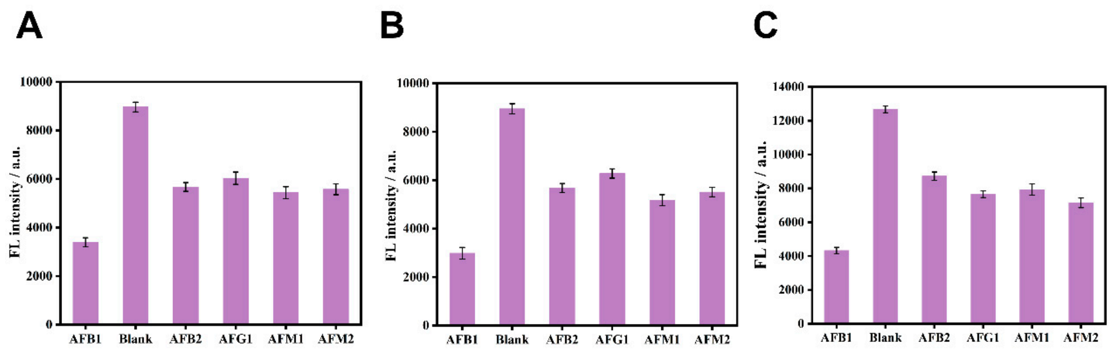

Figure S9. Fluorescence intensity of Zr-LMOFs towards AFB1, AFB2, AFG1, AFM1, and AFM2. (A) Rod-like Zr-LMOF, (B) Prismoid-like Zr-LMOF, (C) Ellipsoid-like Zr-LMOF.

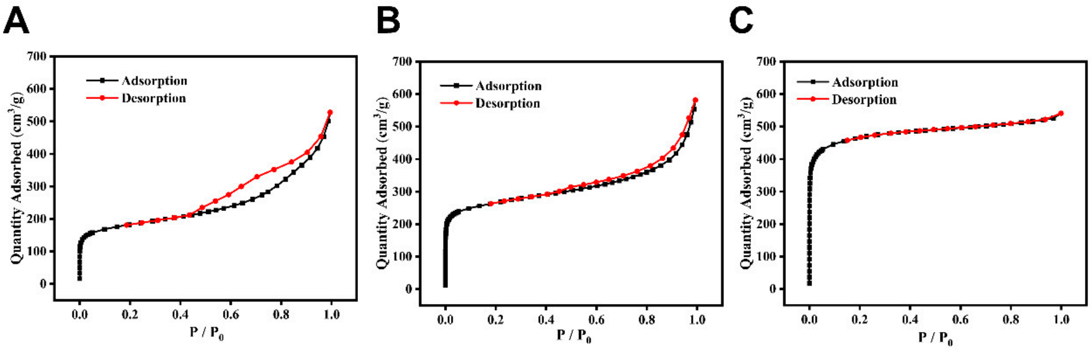

Figure S10. Nitrogen adsorption and desorption isotherms measured at 77.3 K. (A) Rod-like Zr-LMOF, (B) Prismoid-like Zr-LMOF, (C) Ellipsoid-like Zr-LMOF.

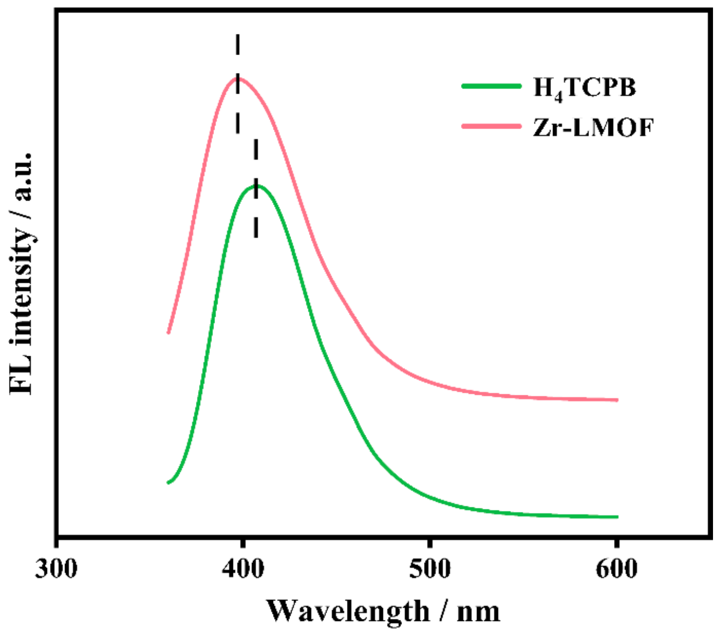

Figure S11. Emission spectra (λ_{em} = 410 nm) of H_4TCPB and Zr-LMOF.

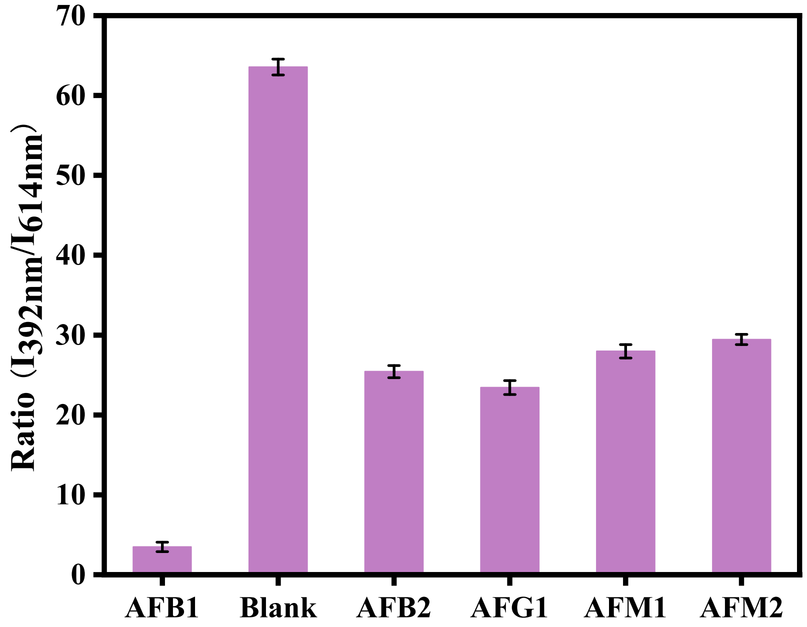

Figure S12. Fluorescence intensity of Zr-LMOF/Eu towards AFB1, AFB2, AFG1, AFM1, and AFM2.

Table S1. Comparison of our method with other sensors for AFB1 detection reported in the literatures.

Detection Method	Linear Range	LOD	Reference
Graphene quantum dots	5.00-800.00 ng mL^{-1}	0.16 nM	[2]
Zr-LMOF	31.20 ppb-15.60 ppm	1.60 ppb	[3]
Al-MOFs	0.05- 9.61 μM	11.67 ppb	[4]
LMOF-241	1.00-16.70 μM	46.00 ppb	[5]
Near infrared fluorescent probe	0.10-38.00 μM	60.00 nM	[6]
Fluorescent nanoprobe	0.1-30 mg kg^{-1}	85.00 μg kg^{-1}	[7]
Rod-like Zr-LMOF	0.005-7.29 μM	12.30 nM	This work
Prismoid-like Zr-LMOF	0.0075-7.29 μM	7.39 nM	
Ellipsoid-like Zr-LMOF	0.005-7.29 μM	5.12 nM	
Zr-LMOF/Eu	0.005-7.29 μM	2.82 nM	

Table S2. Liquid adsorption of AFB1 by Zr-LMOFs.

MOF (100 µg/mL)	Spiked (µM)	Detect in supernate (µM)
Rod-like Zr-LMOF	10.00	8.43
Prismoid-like Zr-LMOF	10.00	7.67
Ellipsoid-like Zr-LMOF	10.00	7.92

Table S3. Zeta potentials of Zr-LMOFs and AFB1.

Sample Name	Zeta Potential (mV) (n=3)
Rod-like Zr-LMOF	-12.50 ± 1.51
Prismoid-like Zr-LMOF	-8.24 ± 0.69
Ellipsoid-like Zr-LMOF	-10.20 ± 0.23
AFB1	-6.90 ± 0.82

Table S4. BET Surface area calculated by the Multi-Point BET method, Pore Volume and Average Pore Size by BJH adsorption, Most Frequent Pore Diameter by HK/SF method of Rod-like Zr-LMOF, Prismoid-like Zr-LMOF, and Ellipsoid-like Zr-LMOF.

**Sample Name**	**BET Surface Area (m^2 g^{-1})**	**Pore Volume (cm^3 g^{-1})**	**Average Pore Size (nm)**	**Most Frequent Pore Diameter (nm)**
Rod-like Zr-LMOF	669.44	0.79	4.71	0.48
Prismoid-like Zr-LMOF	992.10	0.88	3.54	0.46
Ellipsoid-like Zr-LMOF	1773.74	0.82	1.87	0.61

Table S5. HOMO/LUMO energy levels of Zr-LMOF and AFB1.

Mycotoxin/MOF	HOMO/E_{VB} (eV)	LUMO/E_{CB} (eV)	Energy Gap (eV)
AFB1	-11.83	-9.68	2.15
Zr-LMOF	-4.70	-2.12	2.54

Calculation of HOMO/LUMO energy levels for Zr-LMOF

At a preset vacuum level of 4.8 eV, ferrocene was often used as a reference substrate to obtain the initial oxidation potential and reduction potential of other materials [8]. The oxidation potential (E_{ox}) and reduction potential (E_{red}) shown in the ferrocene CV curve are 0.07 eV and 0.21 eV respectively, inset of **Figure 4C**. And the oxidation potential (E_{ox} = 0.63 eV) and the optical band gap (E_g = 2.54 eV) of Zr-LMOF are obtained from the CV curve and plots of $(ahv)^2$ versus energy (hv), respectively, in **Figure 4D** and **4E**. Moreover, calculated according to the following equations (S1) - (S3), the E_{VB} and E_{CB} of Zr-LMOF were estimated to be -4.70 eV and -2.12 eV, respectively.

$$E_{1/2reference} = (E_{ox} + E_{red})/2 \tag{S1}$$

$$E_{VB} = -(4.8 - E_{1/2reference} + E_{ox}) \tag{S2}$$

$$E_{CB} = E_{VB} + E_g \tag{S3}$$

Table S6. Results of our method detecting AFB1 in real samples.

Method	Mode	Sample	Spiked (nM)	Detected (nM)	RSD (N=3, %)	Recovery (%)
ELISA	Colorimetric	Peanut	10	9.7	2.8	97.0
			50	51.4	3.4	102.8
			150	147.2	2.3	98.1
		Wheat	10	11.2	2.6	112.0
			50	48.2	3.1	96.4
			150	150.7	2.7	100.5
Our method	Fluorescence	Peanut	10	11.7	1.3	117.0
			50	51.7	1.8	103.4
			150	136.4	3.1	91.0
		Wheat	10	9.2	2.5	92.0
			50	46.7	4.2	93.4
			150	143.6	2.4	95.7

References

1. Lammert, M.; Reinsch, H.; Murray, C.; Wharmby, M.; Terraschke, H.; Stock, N., Synthesis and structure of Zr (IV)-and Ce (IV)-based CAU-24 with 1, 2, 4, 5-tetrakis (4-carboxyphenyl) benzene. *Dalton Transactions* **2016,** 45, (47), 18822-18826.
2. Singh, A. K.; Sri, S.; Garimella, L. B.; Dhiman, T. K.; Sen, S.; Solanki, P. R., Graphene quantum dot-based optical sensing platform for aflatoxin B1 detection via the resonance energy transfer phenomenon. *ACS Applied Bio Materials* **2022,** 5, (3), 1179-1186.
3. Li, Z.; Xu, X.; Quan, H.; Zhang, J.; Zhang, Q.; Fu, Y.; Ying, Y.; Li, Y., Adsorptive and responsive hybrid sponge of melamine foam and metal organic frameworks for rapid collection/removal and detection of mycotoxins. *Chemical Engineering Journal* **2021,** 410, 128268.
4. Wang, F.; Li, Z.; Jia, H.; Lu, R.; Zhang, S.; Pan, C.; Zhang, Z., An ultralow concentration of Al-MOFs for turn-on fluorescence detection of aflatoxin B1 in tea samples. *Food Chemistry* **2022,** 383, 132389.
5. Hu, Z.; Lustig, W. P.; Zhang, J.; Zheng, C.; Wang, H.; Teat, S. J.; Gong, Q.; Rudd, N. D.; Li, J., Effective detection of mycotoxins by a highly luminescent metal-organic framework. *Journal of the American Chemical Society* **2015,** 137, (51), 16209-16215.
6. Yan, X.; Li, H.; Yan, Y.; Su, X., Selective detection of parathion-methyl based on near-infrared $CuInS_2$ quantum dots. *Food chemistry* **2015,** 173, 179-184.
7. Fahimi-Kashani, N.; Rashti, A.; Hormozi-Nezhad, M. R.; Mahdavi, V., MoS_2 quantum-dots as a label-free fluorescent nanoprobe for the highly selective detection of methyl parathion pesticide. *Analytical Methods* **2017,** 9, (4), 716-723.
8. Xiong, D.; Cheng, J.; Ai, F.; Wang, X.; Xiao, J.; Zhu, F.; Zeng, K.; Wang, K.; Zhang, Z., Insight into the Sensing Behavior of DNA Probes Based on MOF-Nucleic Acid Interaction for Bioanalysis. *Analytical Chemistry* **2023,** 95, (12), 5470-5478.
